# Functional and pharmacological role of the dopamine D_4_ receptor and its polymorphic variants

**DOI:** 10.3389/fendo.2022.1014678

**Published:** 2022-09-30

**Authors:** Sergi Ferré, Annabelle M. Belcher, Jordi Bonaventura, César Quiroz, Marta Sánchez-Soto, Verònica Casadó-Anguera, Ning-Sheng Cai, Estefanía Moreno, Comfort A. Boateng, Thomas M. Keck, Benjamín Florán, Christopher J. Earley, Francisco Ciruela, Vicent Casadó, Marcelo Rubinstein, Nora D. Volkow

**Affiliations:** ^1^ Integrative Neurobiology Section, National Institute on Drug Abuse, Intramural Research Program, National Institutes on Drug Abuse, Baltimore, MD, United States; ^2^ Division of Addiction Research and Treatment, Department of Psychiatry, University of Maryland School of Medicine, Baltimore, MD, United States; ^3^ Pharmacology Unit, Department of Pathology and Experimental Therapeutics, Faculty of Medicine and Health Sciences, Institute of Neurosciences, University of Barcelona, L'Hospitalet de Llobregat, Spain; ^4^ Neuropharmacology & Pain Group, Neuroscience Program, Bellvitge Institute for Biomedical Research, L'Hospitalet de Llobregat, Spain; ^5^ Department of Biochemistry and Molecular Biomedicine, Faculty of Biology, Institute of Biomedicine of the University of Barcelona (IBUB), University of Barcelona, Barcelona, Spain; ^6^ Department of Basic Pharmaceutical Sciences, Fred Wilson School of Pharmacy, High Point, NC, United States; ^7^ Department of Chemistry and Biochemistry, Rowan University, Glassboro, NJ, United States; ^8^ Departament of Physiology, Biophysics and Neurosciences, Centro de Investigación y de Estudios Avanzados del Instituto Politécnico Nacional, Mexico City, Mexico; ^9^ Department of Neurology, Johns Hopkins University School of Medicine, Baltimore, MD, United States; ^10^ Instituto de Investigaciones en Ingeniería Genética y Biología Molecular, Consejo Nacional de Investigaciones Científicas y Técnicas and, Facultad de Ciencias Exactas y Naturales, Universidad de Buenos Aires, Buenos Aires, Argentina; ^11^ National Institute on Drug Abuse, National Institutes of Health, Rockville, MD, United States

**Keywords:** dopamine D4 receptor, polymorphic variants, impulsivity, attention-deficit hyperactivity disorder, restless legs syndrome

## Abstract

The functional and pharmacological significance of the dopamine D_4_ receptor (D_4_R) has remained the least well understood of all the dopamine receptor subtypes. Even more enigmatic has been the role of the very prevalent human *DRD4* gene polymorphisms in the region that encodes the third intracellular loop of the receptor. The most common polymorphisms encode a D_4_R with 4 or 7 repeats of a proline-rich sequence of 16 amino acids (D_4.4_R and D_4.7_R). *DRD4* polymorphisms have been associated with individual differences linked to impulse control-related neuropsychiatric disorders, with the most consistent associations established between the gene encoding D_4.7_R and attention-deficit hyperactivity disorder (ADHD) and substance use disorders. The function of D_4_R and its polymorphic variants is being revealed by addressing the role of receptor heteromerization and the relatively avidity of norepinephrine for D_4_R. We review the evidence conveying a significant and differential role of D_4.4_R and D_4.7_R in the dopaminergic and noradrenergic modulation of the frontal cortico-striatal pyramidal neuron, with implications for the moderation of constructs of impulsivity as personality traits. This differential role depends on their ability to confer different properties to adrenergic α_2A_ receptor (α_2A_R)-D_4_R heteromers and dopamine D_2_ receptor (D_2_R)-D_4_R heteromers, preferentially localized in the perisomatic region of the frontal cortical pyramidal neuron and its striatal terminals, respectively. We also review the evidence to support the D_4_R as a therapeutic target for ADHD and other impulse-control disorders, as well as for restless legs syndrome.

## 1 Introduction

Discovered 30 years ago, the dopamine D_4_ receptor (D_4_R) initially drew attention due to its strong affinity for the atypical antipsychotic clozapine, an affinity found to be significantly higher than for the previously discovered D_1_, D_2_ and D_3_ receptors (D_1_R, D_2_R and D_3_R) (1). At the time, it was assumed that this high affinity could underlie clozapine’s unique clinical efficacy, which led to a very intense search for selective D_4_R antagonists. Unfortunately, the newly discovered D_4_R antagonists that followed were ineffective as antipsychotic drugs in clinical trials and the interest in D_4_R as a target for drug development waned [reviewed in ref. (2)].

Not only the pharmacological, but also the functional significance of the D_4_R has remained the most enigmatic of all the dopamine receptor subtypes. The human D_4_R gene (*DRD4*) displays a high number of polymorphisms in its coding sequence. The most extensive polymorphism is found in exon 3, a region that encodes the third intracellular loop (3IL) of the receptor (3–5). This polymorphism comprises a variable number of tandem repeats of a 48-base pair sequence, from 2- to 11 repeats. The most common polymorphisms contain 2, 4 or 7 repeats (with allelic frequencies of about 8%, 60% and 20%, respectively) (4), which encode a D_4_R with the respective number of repeats of a proline-rich sequence of 16 amino acids (D_4.2_R, D_4.4_R and D_4.7_R) (3–5). *DRD4* polymorphisms have been associated with individual differences in impulse control-related neuropsychiatric disorders, with the most consistent associations found between the gene encoding D_4.7_R and attention-deficit hyperactivity disorder (ADHD) (3, 6–8) and substance use disorders (SUDs) (9). It follows that these significant associations should provide clues about the functional and pharmacological significance of the D_4_R. The main functional and pharmacological properties of the D_4_R and its different D_4_R polymorphic variants have been elucidated only recently with studies addressing the role of receptor heteromerization and the relatively avidity of norepinephrine for D_4_R. This includes the role of pineal D_4_R in the circadian noradrenergic modulation of melatonin synthesis and release (10), as well as the role of D_4_R in the inhibitory dopaminergic modulation of frontal cortico-striatal glutamatergic neurotransmission (11). Here we review the evidence to support the role of frontal cortical D_4_Rs in the moderation of constructs of impulsivity as personality traits. Based on the available evidence, we submit that the D_4_R should be exploited as a therapeutic target for impulse control-related neuropsychiatric disorders (primarily, ADHD), as well as for restless legs syndrome (RLS).

## 2 The D_4_R-modulated frontal-cortico-striatal neuron

D_4_R is highly expressed in the prefrontal cortex of mammals, including rodents and human and non-human primates, particularly in deep layer neurons (1, 12–14). In contrast with other dopamine receptors (D_1_R, D_2_R and in the ventral striatum, D_3_R), striatal mRNA expression of D_4_R is much lower (1, 12). However, immunohistochemical studies using different antibodies against different epitopes of the rodent and human D_4_R produced incongruent results, particularly in relation to its striatal density, indicating a lack of antibody specificity (15–19). Further studies, using a transgenic mouse expressing a fluorescent protein under the transcriptional control of the mouse dopamine D_4_R gene (*Drd4*), confirmed its predominant expression in the deep layer neurons of the prefrontal cortex and its lack of expression in the striatum (20). These results agreed with those obtained using *in situ* hybridization studies and indicate that striatal D_4_Rs are localized in striatal nerve terminals and, most probably in the terminals from glutamatergic neurons originated in the deep cortical layers.

In fact, significant evidence for the existence of D_4_Rs in the prefrontal cortex and the striatum of rats and humans has been obtained by radioligand binding experiments (21–25). The D_4_R labeling strategies included radioligands with significant selectivity for D_4_R or non-selective D_2_-like receptor radioligands in the presence of the D_2_R-D_3_R antagonist raclopride, which demonstrates a specific low affinity for the D_4_R (1). The results showed a disproportionally high density of striatal D_4_R binding sites when compared with the striatal expression of D_4_R mRNA, in agreement with their putative localization in cortico-striatal terminals. This was confirmed with experiments with frontal cortex ablation, which produced a significant reduction of striatal D_4_R binding sites (24).

The neocortex is composed of two major neuronal populations: glutamatergic pyramidal (P) neurons (70-80%) and GABAergic interneurons (20%-30%). There are several subtypes of GABAergic interneurons which have been grouped in three main classes, based on their transcriptional similarities and the expression of selective markers (26, 27). The largest class of cortical interneuron is characterized by the expression of the calcium-binding protein parvalbumin (PV). PV neurons target the perisomatic region of P neurons and control their spiking output. The second class expresses the neuropeptide somatostatin, and these neurons preferentially target the dendritic region of P neurons. And the third class expresses the 5-HT_3a_ serotonin receptor, with a common subclass also expressing the calcium binding protein calretinin and the vasoactive intestinal peptide. These are mainly disinhibitory neurons that preferentially target PV+ and somatostatin+ interneurons. It has been postulated that the three subclasses of GABAergic interneurons establish a local cortical circuit that is critically involved in working memory, by which stimulus tuning of persistent activity arises from the concerted action of widespread inhibition mediated by PV+ interneurons and localized disinhibition of P neurons mediated by calretinin-containing interneurons (27, 28). In non-human primates, most P neurons and nearly half of the GABAergic interneurons express D_4_R. Among the interneurons, D_4_R are particularly expressed in PV+ interneurons (13, 14).

D_4_Rs are therefore positioned to exert a significant modulatory influence on frontal cortico-striatal P neurons. Considering the Gi-coupled D_4_R as mostly inhibitory, those localized in their cortical perisomatic region and striatal terminals should be expected to mediate an inhibitory effect of dopamine, while those localized in PV+ GABAergic interneurons should be expected to produce disinhibition. Nevertheless, a recent electrophysiological study on mouse cortical slices showed that D_4_R activation induces a more complex set of effects, with a direct slow decreasing effect on the excitability of P neurons and a fast and transient increase followed by a delayed decrease of the excitability of PV+ interneurons (29). The initial effect on PV+ interneurons should in fact lead to an initial potent suppression of PFC network activity and output signal. The effect of the delayed decrease of the excitability of PV+ interneurons on P neurons should be masked by the direct D_4_R-mediated decrease excitability of P neurons. In summary, activation of frontal cortical D_4_R should mostly keep a low output signal of the PFC network (29). In addition, a recent study using a combined optogenetic-microdialysis technique demonstrated the ability of D_4_Rs localized in frontal cortico-striatal terminals to exert a significant inhibitory role of striatal glutamate release (11). Overall, these studies suggest that D_4_Rs are instrumental for the dopamine-mediated functional inhibition of frontal cortico-striatal neurotransmission, in agreement with the hyperexcitability observed on frontal cortical P neurons in D_4_R-deficient mice (30). However, as reviewed below, several studies indicate a more complex picture, where D_4_Rs can also directly mediate the effects of norepinephrine and indirectly modulate the function of adrenoceptors and other dopamine receptor subtypes by heteromerization.

## 3 Lessons from the pineal gland. D_4_R heteromerization and D_4_R as a target for norepinephrine

Apart from the frontal cortex, D_4_Rs are highly expressed in the retina (31) and by the pinealocytes of the pineal gland (32), which main function is the circadian secretion of the hormone melatonin. This circadian control is mediated by a neuronal circuit that includes the suprachiasmatic and paraventricular nuclei of the hypothalamus, the intermediolateral column of the spinal cord and the superior cervical ganglion, which sends noradrenergic afferents to the pineal gland (33, 34). Darkness-induced norepinephrine release in the pineal gland activates pinealocyte Gs-coupled β_1_ and Gq-coupled α_1B_ adrenergic receptors (β_1_R and α_1B_R), which promotes the synthesis and release of melatonin and its precursor serotonin (33, 34). Several experimental observations indicate that D_4_Rs play a fundamental role in the circadian adrenergic control of melatonin synthesis by pinealocytes and that this role depends on their circadian expression, their heteromerization-dependent ability to inhibit β_1_R and α_1B_R function and their ability to bind and be activated by norepinephrine (10, 32, 35).

D_4_R expression in the retina and pineal gland varies significantly in a circadian manner, being particularly elevated during the second half of the dark period (32). This increased expression is under the control of pineal β_1_R and α_1B_R and requires thyroid hormone (32). We found evidence indicating that D_4_R can form heteromers with both β_1_R and α_1B_R in mammalian transfected cells and in rat pinealocytes, and that the expression of β_1_R-D_4_R and α_1B_-D_4_R heteromers follows the circadian expression of D_4_R, being maximal at sunrise (10). In these heteromers, activation of D_4_R leads to a significant decrease in the ability of its partner adrenoceptor to signal (10), apparently suggesting that, in the pineal gland, dopamine inhibits the effect of norepinephrine. However, there is no clear evidence for a functional significant dopamine release in the pineal gland, where the catecholaminergic input is largely noradrenergic, the noradrenergic afferents from the superior cervical ganglion (33, 34).

A similar situation can be found in the cerebral cortex, particularly of rodents, which receives dense and widespread noradrenergic innervation, whereas dopaminergic terminals are restricted to the prefrontal cortex (36, 37). However, dopamine receptors are expressed throughout the cortex, and their localization exceeds that of the dopaminergic terminals (38–41). It has therefore been suggested that dopamine is either a co-neurotransmitter in noradrenergic neurons (42) or that there is a long-range volume-transmission of catecholamines (43). In fact, asynaptic varicosities represent most cortical norepinephrine releasing sites (37). A more parsimonious mechanism is the possibility that endogenous norepinephrine can also be an endogenous ligand of D_4_R. Thus, as demonstrated with *in vitro* experiments, norepinephrine binds and activates D_4_Rs at submicromolar concentrations (35, 44–46), up to ten times higher than the concentration able to activate β_1_R or α_1B_R in pineal cell preparations or pineal tissue (47, 48). Neverthelss, the potency of norepinephrine at activating pineal D_4_Rs needs to be determined. Altogether, a very plausible mechanism by which pineal D_4_Rs control melatonin synthesis and release emerges: at the beginning of the dark period, the initial noradrenergic activation of the pineal gland targets β_1_R and α_1B_R, favoring a progressive increase in melatonin synthesis, but also D_4_R expression; whereas at the end of the dark period, the increased expression of D_4_Rs leads to the formation of β_1_R-D_4_R and α_1B_-D_4_R heteromers, which allows norepinephrine to preclude β_1_R and α_1B_R signaling within the heteromer, thus dwindling melatonin synthesis and release (10).

## 4 The enigmatic role of *DRD4* polymorphic variants. GPCR oligomerization comes to the rescue

The question of the functional significance of the different receptor isoforms encoded by *DRD4* polymorphisms has remained enigmatic until recently. Those include electrophysiological and biochemical experiments in frontal cortical slices from D_4_R knockout mice with rescued expression of human D_4.4_R and D_4.7_R by viral transduction (49, 50), and immunohistochemical and *in vivo* optogenetic-microdialysis experiments in D_4.7_R knock-in mice expressing a humanized D_4_R with the 3IL of the human D_4.7_R (11). Electrophysiological experiments in frontal cortical slices from D_4_R knockout mice showed an increased ability of a D_4_R agonist to suppress network bursts and NMDA receptor-mediated excitatory postsynaptic currents from P neurons after viral transduction of human D_4.7_R, as compared with human D_4.4_R receptor cDNA (49, 50). Interestingly, biochemical experiments in the same slice preparations revealed that these differences correlated with a more profound D_4_R agonist-mediated downregulation of NMDA receptor surface expression (i.e., NR1 subunit) in frontal cortical slices virally transduced with D_4.7_R than with D_4.4_R (49). The *in vivo* experiments in D_4.7_R knock-in mice showed a blunting of methamphetamine-induced cortical activation and ontogenetically and methamphetamine-induced frontal cortico-striatal glutamate release (11). In summary, these studies showed a pronounced gain of function of D_4.7_R, as compared to D_4.4_R, in its ability to mediate the inhibitory influence of dopamine on frontal cortico-striatal neurotransmission.

The next enigma to solve was the mechanism behind the functional differences of the products of *DRD4* polymorphisms. Thus, in mammalian transfected cells, D_4.2_R, D_4.4_R and D_4.7_R did not show clear pharmacological differences in response to endogenous or exogenous ligands, although an earlier study seemed to indicate that D_4.7_R signals with less efficiency than D_4.4_R (51). Nevertheless, by using a functional bioluminescence resonance energy transfer (BRET) technique, we could not find significant differences in the ability of the D_4.2_R, D_4.4_R or D_4.7_R polymorphic variants to promote dopamine-induced activation of any of the five Gi/o protein subtypes (35). Importantly, differences emerged when studying their ability to form heteromers, more specifically with the Gi-coupled D_2_R and α_2A_R, preferentially localized in the perisomatic region and nerve terminals of prefrontal cortical P neurons, respectively (52–54).

Apart from D_4_Rs, a significant proportion of frontal cortical P neurons from layer V also express D_2_Rs (in monkeys, about 75% and 55%, respectively) (14). Furthermore, *in vitro* experiments in striatal slices (52) and *in vivo* optogenetic-microdialysis experiments in rodents (11, 55) have provided evidence for the presence of functional D_2_R and D_4_R receptors in cortico-striatal glutamatergic terminals. This provides the framework for the existence of D_2_R-D_4_R heteromers in striatal terminals and, possibly, in the perisomatic region of P neurons. The first studies on heteromers of the D_4_R polymorphic variants, based on BRET and co-immunoprecipitation techniques in transfected mammalian cells, suggested that the D_4.7_R establishes weaker intermolecular and functional interactions with the D_2_R (both isoforms, D_2L_R and D_2S_R) than the D_4.4_R (52, 56, 57). At the functional level, co-transfection of D_2_Rs with D_4.4_Rs, but not D_4.7_Rs, led to an increase in MAPK signaling (52, 57), which would seem opposite to the expected D_4.7_R gain of function. Nevertheless, a dependence on heteromerization of these differences in MAPK signaling was not clearly established. On the other hand, a more recent study using the complemented donor acceptor bioluminescence resonance energy transfer (CODA-RET) technique demonstrated a D_4.7_R heteromerization-dependent gain of function of the D_2_R (53).

CODA-RET is a BRET assay that allows the measurement of ligand-induced changes in the interaction between G protein-coupled receptors (GPCRs) forming specific homomers or heteromers with transducer proteins, including G proteins (58). In the CODA-RET assay, two complementary halves of a bioluminescent chromophore, such as *Renilla* Luciferase are separately fused to two different receptor molecules putatively able to oligomerize and a fluorescent chromophore (such as yellow fluorescent protein) can be fused to a G protein subunit. Ligand-induced changes in CODA-RET measurements imply, first, a successful complementation of *Renilla* Luciferase and, therefore, oligomerization of the corresponding GPCR units. Second, although CODA-RET does not provide estimates of the degree of oligomerization, such as the affinity of the intermolecular interactions between GPCR subunits or the proportion of subunits forming oligomers, this technique does allow a qualitative measure of the ability of a specific endogenous or exogenous ligand to activate a GPCR homodimer or heterodimer and signal through a specific G protein (58, 59). Thus, using CODA-RET, we were able to disclose, for the first time, a different qualitative profile of several D_2_-like receptor ligands for D_4.4_R and D_4.7_R, but only when forming heteromers with D_2_R or α_2A_R (53, 54).

**Figure 1:**
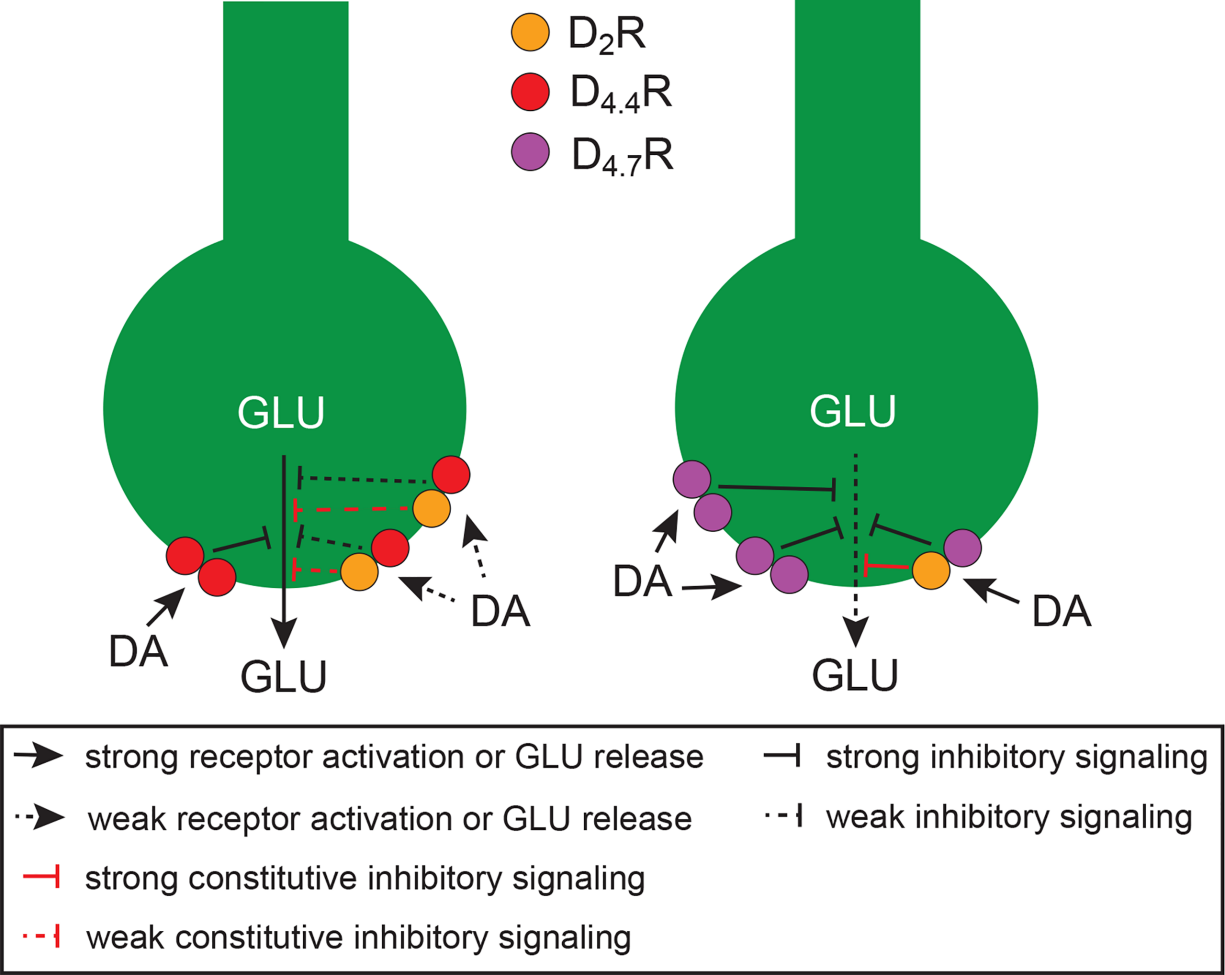
Schematic representation of cortico-striatal glutamatergic terminals and their modulatory D_4.4_Rs (left terminal) and D_4.7_Rs (right terminal), which form heteromers with D_2_Rs. Heteromerization of D_2_R with D_4.7_R promotes a gain of function of the dopaminergic-mediated inhibition of glutamate (GLU) release, as compared with heteromerization with D_4.4_R, since it increases the potency for dopamine (DA) and the constitutive activity of the D_2_R. In addition, the population of D_4.7_Rs not forming heteromers with D_2_Rs is larger than with D_4.4_Rs, which increases the potency of DA to inhibit GLU release, because of the higher affinity of DA for D4Rs versus D2Rs. The scheme is based on results obtained from experiments in mammalian transfected cells and from *in vitro* and *in vivo* experiments in rodents (see text).

Using CODA-RET, we found two possible mechanisms for the D_4.7_R gain of function as mediator of an inhibition of frontal cortico-striatal neurotransmission (**Figure 1**): first, a specific increase in the potency of dopamine for the D_2_R-D_4.7_R, but not for D_2_R-D_4.4_R heteromer, as compared with the D_2_R homomer (53); second, a divergent decrease or increase in the constitutive activity of D_2_R when it was forming D_2_R-D_4.4_R or D_2_R-D_4.7_R heteromers, respectively (53). In addition, a fraction of D_4_R should not be expected to form heteromers, but homomers, which should be more prevalent with D_4.7_R than with D_4.4_R. In fact, as compared with D_4.4_R, not only D_4.7_R seems to be less able to heteromerize with D_2_R (52, 56, 57), but it also seems to be more able to homomerize (56). Since CODA-RET experiments also demonstrated a significantly higher potency of dopamine for D_4.4_R-D_4.4_R and D_4.7_R-D_4.7_R homomers than for D_2_R-D_4.4_R and the D_2_R-D_4.7_R heteromers (53), the expected larger population of D_4.7_R-D_4.7_R versus D_4.4_R-D_4.4_R homomers should represent a third mechanism involved in the gain of function of D_4.7_R (**Figure 1**).

**Figure 2:**
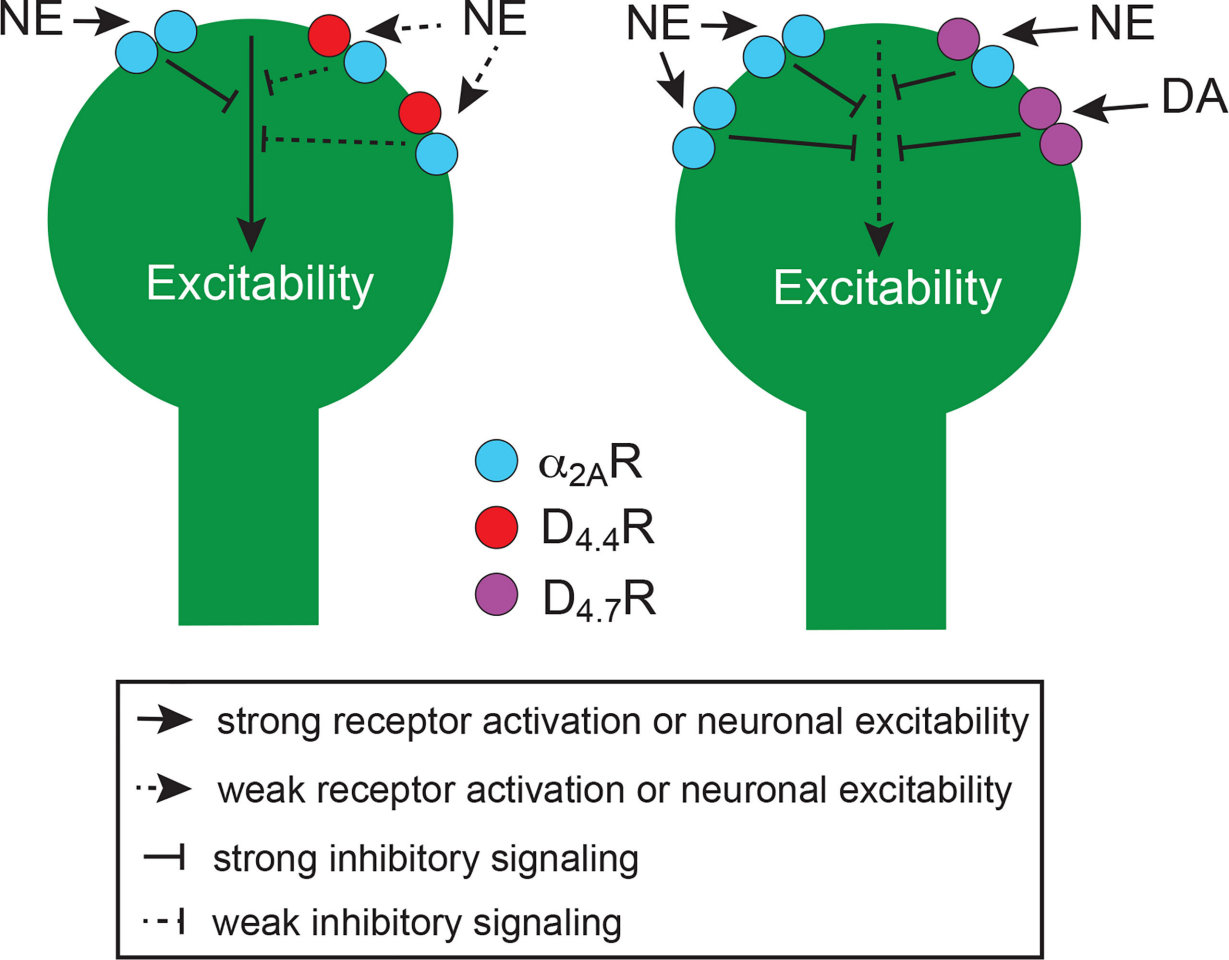
Schematic representation of the perisomatic region of frontal cortical P neurons and their modulatory D_4.4_Rs (left neuron) and D_4.7_Rs (right neuron), which form heteromers with α_2A_Rs. Heteromerization of α_2A_R with D_4.7_R promotes a gain of function of the noradrenergic-mediated inhibition of neuronal excitability, as compared with heteromerization with D_4.4_R, since it increases the potency of norepinephrine (NE). In addition, high concentrations of NE promote a decrease in the potency of NE in the α_2A_R-D_4.4_R but not in the α_2A_R-D_4.7_R heteromer (see text). Furthermore, the population of D_4.7_Rs not forming heteromers with α_2A_R is larger than with D_4.4_Rs, which also increases the population of α_2A_R not forming heteromers and therefore the potency of NE to decrease neuronal excitability. The increased proportion of D_4.7_Rs not forming heteromers additionally increases the ability of DA to decrease neuronal excitability. The scheme is based on results obtained from experiments in mammalian transfected cells and from *in vitro* experiments in rodents (see text).

In view of the involvement of D_4_R and α_2A_R in impulsivity and ADHD (see next section), their concurrent localization in frontal cortical P neurons (see below) and the demonstrated ability of D_4_R to form functional heteromers with other adrenoceptors in the pineal gland (see above), we investigated the possible existence of functionally significant α_2A_R-D_4.4_R and α_2A_R-D_4.7_R heteromers in the brain. Using several biophysical and biochemical techniques and using heteromer-specific disruptive peptides, we demonstrated the ability of both D_4.4_R and D_4.7_R to heteromerize with α_2A_R both in mammalian transfected cells and in the mouse cerebral cortex (54). The results of BRET experiments indicated that, akin to D_2_R-D_4_R heteromers, the D_4.7_R variant was less able to heteromerize with α_2A_R than D_4.4_R. Furthermore, results from radioligand-binding, CODA-RET and signaling experiments indicated that heteromerization with D_4.7_R, but not with D_4.4_R, increases the potency of norepinephrine at activating α_2A_R. Furthermore, D_4.4_R, but not D_4.7_R activation, allosterically inhibited α_2A_R-mediated signaling in their respective heteromers (54). Thus, dopamine should be able to promote a significant inhibitory effect of α_2A_R signaling though α_2A_R-D_4.4_R, but not α_2A_R-D_4.7_R heteromers. Furthermore, as elaborated below, D_4_R can also be activated by endogenous norepinephrine in the cerebral cortex, and high concentrations should determine a significant inhibition of α_2A_R signaling by the α_2A_R-D_4.4_R, but not by the α_2A_R-D_4.7_R heteromer. If the main functional output of α_2A_R-D_4_R heteromers is a decrease in excitability of P neurons (see below, section 6) this could provide an additional mechanism for the gain of function of D_4.7_R in its inhibitory control of frontal cortico-striatal neurotransmission (**Figure 2**).

## 5 D_4_R as a moderator of the personality traits action and choice impulsivity

The most popular models of personality are the Big-Three and the Big-Five models, operationalized by the Multidimensional Personality Questionnaire (MPQ) (60) and the NEO Personality Inventory-Revised (NEO-PI-R) psychometric tests (61), respectively. The MPQ measures three orthogonal traits: positive emotionality (*PEM*), negative emotionality (*NEM*) and constraint (*CON*) (60, 62). *PEM* and *NEM* incorporate dispositions toward positive and negative emotions, respectively, and are linked conceptually to the brain systems underlying appetitive-approach and defensive-withdrawal behaviors. Constraint (*CON*) encompasses dimensions related to behavioral restraint, the opposite end of which implies disinhibition. NEO-PI-R (61) measures neuroticism (*N*), extraversion (*E*), openness (*O*), agreeableness (*A*) and conscientiousness (*C*). *N* and *E* highly correlate with *NEM* and *PEM* respectively, generally constituting the same personality constructs (63, 64). *O* captures interest toward experience, and *A* implies an empathic personality. Finally, *C* is a spectrum of constructs that describes individual differences in the propensity to be self-controlled, responsible to others, hardworking, orderly, and rule abiding (65).

Impulsivity is defined as a predisposition toward rapid, unplanned reactions to internal or external stimuli with little regard for the negative consequences to the individual or others (66). Impulsivity has been decomposed into “rapid-response” or “action” impulsivity, and “cognitive” or “choice” impulsivity (67–69). Action impulsivity (*AI*) is defined as a diminished ability to inhibit prepotent responses, or a failure of volitional motor inhibition or disinhibition (68). Choice impulsivity (*CI*) implies a tendency to accept small immediate or likely rewards at the expense of large delayed or unlikely rewards (69). Excessive *CI* overlaps conceptually with impairment in decision-making and particularly with temporal or delay discounting (70). Delay discounting is the phenomenon by which a delayed outcome of a choice reduces the subjective value of a reward and constitutes an operational measure of the degree of *CI* (71, 72). We have previously maintained that *AI* constitutes the same concept as strong disinhibition, the opposite end of the personality trait *CON* (9), and that the trait *C* encapsulates both dimensions of impulsivity (*AI* and *CI*) (73). In fact, a significant correlation between measures of *CON* and *C* has been reported (63, 64). We have also argued that the same as *AI*, *CI* fulfills the criteria to be considered as a personality trait (73). The substantial overlap of several of these personality traits with maladaptive behaviors and specifically, with mental health disorders, justifies the sustained search for their neural underpinnings as therapeutic targets. But this has been a significant challenge for the field of neuropsychiatry.

The ‘endophenotype’ concept has provided an invaluable approach for the identification of genes that predispose or indemnify individuals from mental and psychiatric disorders. The endophenotype concept is understood as simpler clues to genetic underpinnings than the disease syndrome itself and involves the genetic analysis of any of a variety of biological markers (cognitive, neurophysiological, anatomical, biochemical, etc.) of the disease. The concept promotes the view that psychiatric diagnoses can be decomposed or deconstructed into more tractable intermediate phenotypes by virtue of their assumed proximity to the genetic antecedents of the disease (74, 75). Based on the results of studies that have linked the structure of psychopathology to the structure of personality, as defined by the MPQ or NEO-PI-R (76), we argued previously that specific personality traits constitute endophenotypes of mental health disorders, such as SUD (9).

**Figure 3:**
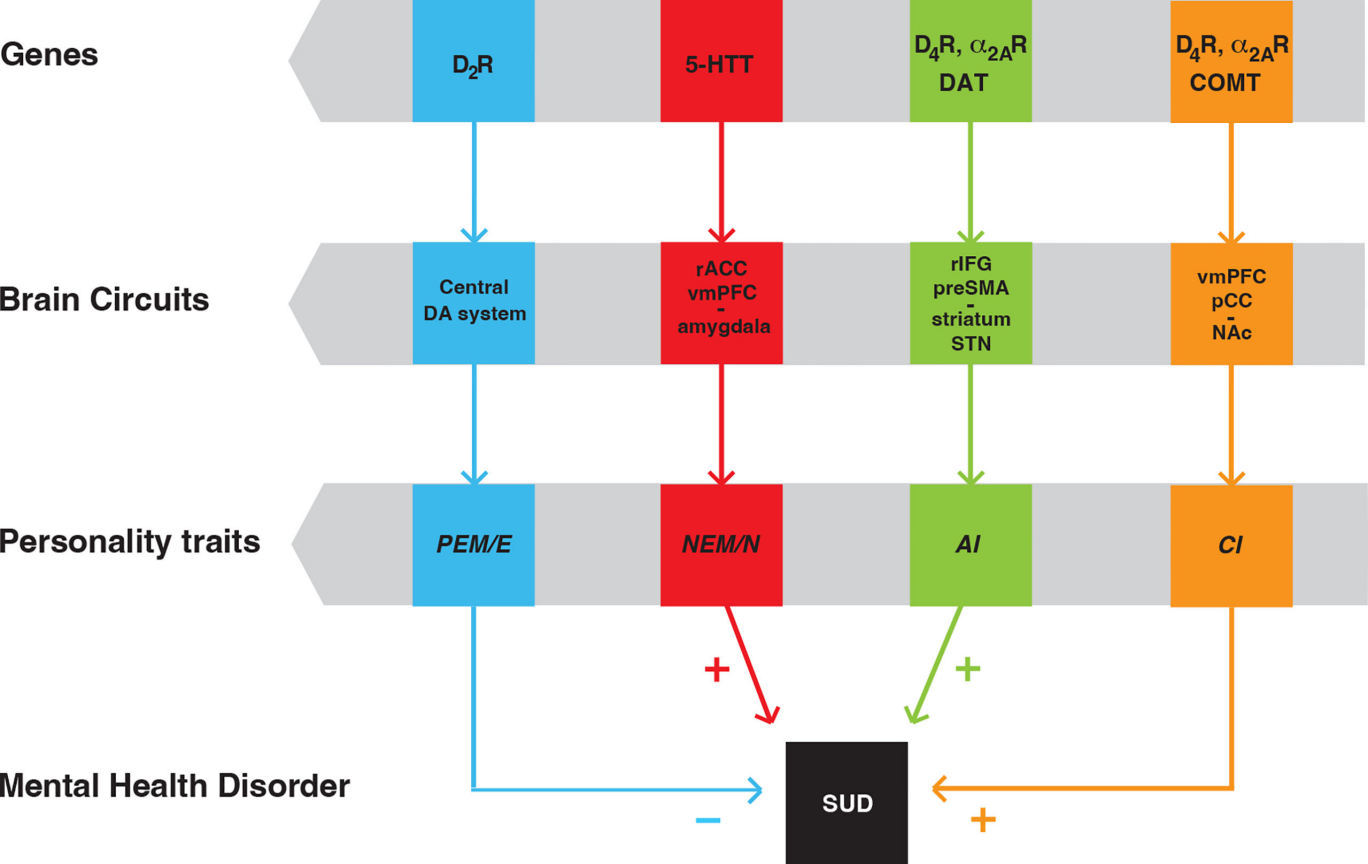
Scheme of personality traits as endophenotypes of SUD, with their linkage to specific brain circuits and genes. Positive emotionality/extroversion (*PEM/E*) is modulated by the central dopaminergic (DA) system and is moderated by the D_2_R gene. Negative emotionality/neuroticism (*NEM/N*) is modulated by a circuit that involves the right anterior cingulate cortex (rACC), ventromedial prefrontal cortex (vmPFC) and the amygdala and is moderated by the gene of the serotonin transporter (5-HTT). Action impulsivity (*AI*) is modulated by a circuit that includes the pre-supplementary motor area (preSMA), the right inferior frontal gyrus (rIFG), the striatum and the subthalamic nucleus (STN). Choice impulsivity (*CI*) is modulated by a circuit that includes the ventromedial prefrontal cortex (vmPFC), the posterior cingular cortex (pCC) and the nucleus accumbens (NAc). *AI* and *CI* are moderated by the genes of the dopamine transported (DAT) and the enzyme cathecol-*O*-methyltransferase (COMT), respectively. In addition, *AI* and *CI* are both moderated by the genes of D_4_R and α_2A_R. “+” and “-” indicate that individuals with low *PEM*, high *NEM*, high *AI* and high CI are most vulnerable (least resilient) to develop SUD [modified from ref (71)].

We initially identified *PEM*/*E* (from the MPQ and NEO-PI-R assessments, respectively), *NEM*/*N* (again, from the two assessments, respectively), and *CON* (the inverse measure of *AI*), as tied to specific brain circuits and genes (9). *PEM/E* is modulated by the function of the central dopaminergic system and is moderated by the D_2_R gene. *NEM/N* is modulated by the glutamatergic outputs from the right anterior cingulate cortex and ventromedial prefrontal cortex to the amygdala and insula and is moderated by the serotonin transporter gene. *AI* is modulated by a circuit including the glutamatergic neurons arising in the pre-supplementary motor area and right inferior frontal gyrus and innervating the dorsal striatum and the subthalamic nucleus and is moderated by the genes of the D_4_R and the dopamine transporter (9) (**Figure 3**). Individuals with low *PEM/E*, high *NEM/N* and high *AI* would be most vulnerable (least resilient) to develop SUD. Conversely, individuals with high *PEM/E*, low *NEM/N* and low *AI* would be least vulnerable (most resilient) to SUD (9).

To our initial analysis, we added *CI* as an additional personality trait, modulated by a circuit including glutamatergic neurons from the ventromedial prefrontal cortex and the posterior cingular cortex that innervate the ventral striatum (nucleus accumbens) and, as with *AI*, is also moderated by the D_4_R gene, and by the gene of the enzyme catechol-*O*-methyltransferase (73) (**Figure 3**). The fact that D_4_R moderates both *AI* and *CI* provide a clue for the apparent orthogonality of the *C* trait, as it encapsulates both traits (see above), which constitute endophenotypes for ADHD (76–80) and SUD (9, 73). Not surprisingly, as for SUD, low *C* is a consistent finding in ADHD (81), which if left untreated, constitutes a risk factor for SUD (82–84).

The same as for the D_4_R gene, polymorphisms of the α_2A_R gene may confer vulnerability to developing ADHD as well as symptoms of impulse control disorders (85, 86). However, a large meta-analysis did not find a consistent significant association (87). Yet, when studied at the intermediate phenotype level, as an endophenotype, a clear significant association was established between α_2A_R gene polymorphisms and *AI* (88). Additionally, the α_2A_R agonist guanfacine, which is currently used in the symptomatic treatment of ADHD (89), significantly decreases delay discounting in nonhuman primates (90). Our recent study on α_2A_R-D_4_R heteromers brings together two key receptors involved in the pathogenesis and treatment of ADHD, since α_2A_R-D_4_R heteromers represent a significant population of both catecholaminergic receptors in the mouse cerebral cortex (54).

## 6 D_4_R antagonists as a plausible treatment for ADHD and other impulse-control disorders

Apart from the association with D_4.7_R, several other preclinical and clinical findings converge on the involvement of D_4_R and its heteromers in ADHD and other impulse-control disorders and, therefore, on its possible utility as a therapeutic target for those disorders. At the preclinical level, several animal models of ADHD with varied face, construct, and predictive validity have been developed, particularly in rodents (91). The complexity of the clinical symptoms and pathology of ADHD has been very challenging for the development of those models and, unfortunately, the lack of knowledge about the etiology and pathogenetic mechanisms of the disorder hampers their construct validity.

The most consistent pathogenetic finding in ADHD is a frontal cortical hypoactivity (92, 93), for which there is not yet a clearly accepted explanation. Although it might seem counterintuitive that a decrease and not an increase in cortico-striatal glutamatergic transmission is associated with impulsivity and ADHD, this could probably be explained by considering the well-established differential effects of the activation of the direct and indirect striatal efferent pathways. Classically, the direct and indirect pathways have been conceptualized as gas and brake pedals of the output signals of the basal ganglia (“Go” and “NoGo” pathways), respectively (94). It therefore seems that a sufficient decrease in the activation of the indirect pathway (releasing the gas pedal) determines an increase in the basal ganglia output, irrespective of a concomitant decreased activation of the direct pathway. Considering a more cognitive conceptualization, which includes the processing of information of relevant and irrelevant stimuli (95), a blunted cortico-striatal neurotransmission affecting the activity of both the direct and indirect striatal efferent pathways should decrease their respective ability to increase the reactivity to reward-related stimuli and to suppress the reactivity to nonrewarded- or aversive-related stimuli. As previously proposed (11), the outcome should be an increased “interest” for irrelevant stimuli and a reduced inhibition of irrelevant responses, which could be important in explaining not only the impulsivity, but also the attentional deficit of ADHD.

Genetic manipulations of the D_4_R in the experimental animal have provided significant correlative information supporting the role of a decrease in striatal glutamatergic transmission in ADHD. As mentioned before, D_4_R-knockout mice showed hyperexcitability of frontal cortical P neurons (30), while the gain of function provided by D_4.7_R induced the opposite effect, with a decrease in cortico-striatal glutamatergic transmission (11). In addition, one of the classical animal models of ADHD, the rodent with neonatal lesions with 6-OH-dopamine, showed an ADHD-like phenotype, including locomotor hyperactivity, paradoxical hypolocomotor response to amphetamine and methylphenidate and poor behavioral inhibition, which was counteracted by genetic or pharmacological blockade of the D_4_R (96, 97). Furthermore, it was also shown that locomotor hyperactivity in 6-OH-dopamine-lesioned rats correlate with increases in the striatal density of D_4_R (97).

The 6-OH-dopamine ADHD rodent model also demonstrated predictive value, since apart from amphetamine and methylphenidate, selective norepinephrine uptake inhibitors were effective at counteracting locomotor hyperactivity (98). In fact, apart from amphetamine and methylphenidate, the inhibitor of the norepinephrine transporter atomoxetine and the α_2A_R agonists guanfacine and clonidine are the most accepted pharmacological treatments for ADHD (89). In the cortex, α_2A_Rs are preferentially localized postsynaptically, in P neurons of the deep layers (99, 100), therefore, potentially co-localized with D_4_R. In fact, as mentioned before, we have recently provided experimental evidence indicating that α_2A_R-D_4_R heteromers represent a significant population of both receptors in the mouse cerebral cortex (54). Two different and opposite neuronal effects, both dependent on Gi protein-mediated decrease in cAMP formation, have been described upon activation of cortical postsynaptic α_2A_R: an excitatory effect, dependent on the inactivation of hyperpolarization-activated cyclic nucleotide-gated (HCN) channels (101), and an inhibitory effect, dependent on the inactivation of AMPA receptors (102, 103). These results indicate the existence of two different functional populations of α_2A_R. It is assumed that the excitatory effect mediates the therapeutic effect of α_2A_R agonists, by counteracting the cortical frontal hypoactivity of ADHD (100). It was then suggested that the inhibitory effect would be mediated by a different population of α_2A_R, which could provide a protective mechanism upon overstimulation by high levels of norepinephrine released under stress conditions (102).

It is plausible, and experimentally testable, that D_4_Rs mostly heteromerize and modulate the population of α_2A_Rs that mediate the inhibitory effect of P neuronal function. This could explain at least part of the protective effect of D_4.4_R in ADHD, as well as the D_4.7_R-mediated increased vulnerability to develop ADHD. Thus, the same as with pineal β_1_R-D_4_R and α_1B_-D_4_R heteromers, the cortical α_2A_R-D_4.4_R heteromer could function as a norepinephrine concentration-sensing device, where high concentrations activate D_4.4_R and counteract the effect of the activation of the α_2A_R in the heteromer, diminishing the α_2A_R-mediated inhibitory effect on P neuronal function. On the other hand, the increase in the potency of norepinephrine for α_2A_R in the α_2A_R-D_4.7_R heteromer would facilitate the α_2A_R-mediated inhibitory effect on P neuronal function (**Figure 2**). As in cortico-striatal terminals, activation of D_4_R localized in the perisomatic region of P neurons not forming heteromers (or possible forming heteromers with D_2_R) should directly promote an inhibition of the activity of P neurons (**Figure 2**). D_4_R antagonists should then be considered as possible therapeutic targets for ADHD, as predicted by the positive results obtained in rodents with neonatal 6-OH-dopamine lesions (96, 97), specially, when considering their additional ability to counteract the dopamine-mediated inhibition of glutamate release by cortico-striatal terminals.

However, a randomized, double-blind, crossover study with the selective D_4_R antagonist L-745,870 (also named MK-0929) in adults with ADHD did not demonstrate a significant effect over placebo (104). To our knowledge, this is the only published study addressing the possible clinical efficacy of D_4_R antagonists in ADHD. Obvious limitations, such as the small number of patients, short duration of each treatment period and lack of quantitative cognitive assessments of inhibitory control and other executive functions cannot be ignored. Importantly, D_4.7_R polymorphism characterization was not included in this study, which may also explain the negative findings. Apart from the above reviewed preclinical role of D_4_R as a very significant mediator of the effect of dopamine and norepinephrine on the function of frontal cortical P neuronal function, there is the unequivocal association of D_4.7_R to ADHD. It would therefore be important to carry out more clinical studies with D_4_R antagonists, which should also include addressing their ability to modify the underlying endophenotypes *AI* and *CI* (9, 73).

## 7 D_4_R agonists as plausible treatment of restless legs syndrome

The term akathisia is used to define an urgent need to move. Thus, the primary component of akathisia is a sensory experience which acts as a “drive” or “motivational state” that compels the subject to move. This is objectively perceived by an observer as restlessness or motor hyperactivity (105). Akathisia is also implicit in the description of the symptoms of the very prevalent sensorimotor disorder RLS, where, more often, the sensory experience is an urgent need to specifically move one’s legs (106). Brain iron deficiency (BID), more often without concomitant peripheral iron deficiency, is recognized as the main initial pathogenetic mechanism in RLS (107, 108). BID seems then to trigger a series of pathogenetic mechanisms, including an increase in the motor-cortical and thalamic excitability, conceptualized as a hyperglutamatergic state, that seems to underly RLS symptomatology (108).

BID in rodents is a well-accepted animal model of RLS, which can be induced by providing a severe iron-deficient diet during the postweaning period. The model has both construct and face validity since it recapitulates several biochemical and behavioral findings of RLS (109, 110). Using the optogenetic-microdialysis method, an increase in the sensitivity of cortico-striatal terminals to release glutamate could be demonstrated in the rat with BID, since a lower frequency of optogenetic stimulation was necessary to induce striatal glutamate release as compared to controls (55). This increased sensitivity seems to be related to a BID-induced alteration in the expression of adenosine receptor subtypes in the cortico-striatal glutamatergic terminals, with a downregulation of adenosine A_1_ receptors (A_1_Rs) and a relative upregulation of adenosine A_2A_ receptors (A_2A_Rs) (111, 112). Using the optogenetic-microdialysis method, evidence was also obtained for the ability of the equilibrative nucleoside transporter dipyridamole, which increases the extracellular levels of adenosine, to significantly inhibit cortico-striatal glutamate in control rats and in rats with BID (113). The results of these experiments indicated a possible therapeutic effect of dipyridamole, which was recently demonstrated by two clinical studies, an open trial, and a randomized, placebo-controlled crossover study (114, 115). These studies provided a new therapeutic approach for RLS and significantly validated the cortico-striatal glutamatergic terminals as targets for the treatment of RLS.

Using the same model, perfusion of the most prescribed drugs in RLS, pramipexole, ropinirole and gabapentin, all counteracted the ability of optogenetic stimulation to induce glutamate release, both in controls and in rats with BID (55). By binding to the α_2_δ subunit of voltage-dependent calcium channels localized in glutamatergic terminals, gabapentin reduces their function and trafficking, therefore decreasing striatal glutamate release (116). Pramipexole and ropinirole are non-selective D_2_-like receptor agonists with a slightly higher affinity for D_3_R. It has therefore been suggested that their therapeutic effect is related to their preferential affinity for D_3_R (117). However, the effect of pramipexole on the optogenetically induced glutamate release was not antagonized by a selective D_3_R antagonist, but by D_2_R and D_4_R antagonists (55). These results, therefore, provided a significant support to the key mediation of D_2_R and D_4_R in the local striatal inhibitory control of dopamine on cortico-striatal glutamate release.

The predictive value offered by the conceptual framework of an increased sensitivity of cortico-striatal terminals in RLS points to D_4_R agonists as a possible new treatment for RLS. This could possibly avoid secondary effects of the currently used dopaminergic compounds, which very commonly lead not only to disappearance of their therapeutic effect, but to an increase in the RLS symptoms, known as “augmentation” (118). Thus, it is conceivable that augmentation is secondary to activation of postsynaptic dopamine receptors, similar to the mechanism involved in L-DOPA-induced dyskinesia, a common complication of the treatment with dopaminergic compounds in Parkinson’s disease (119).

## 8 Conclusions

We reviewed the evidence conveying a significant role of D_4_R in the dopaminergic and noradrenergic modulation of the frontal cortico-striatal pyramidal neuron, with implications for the moderation of constructs of impulsivity as personality traits. We also reviewed the evidence strongly supporting that these D_4_Rs should be exploited as therapeutic targets for ADHD and other impulse-control disorders and for RLS. Special emphasis was placed on the concept of receptor heteromerization, which has played a fundamental role in the understanding of D_4_R function and in the understanding of the different functional differences between D_4_R polymorphic variants.

Particularly striking is the fact that the most common polymorphic variants, D_4.4_R and D_4.7_R (with allelic frequencies of about 60% and 20%, respectively) (4), confer significantly different functional and pharmacological properties to α_2A_R-D_4_R and D_2_R-D_4_R heteromers, which mediate a dopamine- and norepinephrine-dependent fine-tune modulation of the frontal cortico-striatal glutamatergic neuronal function. This can explain the differential effect of D_4_R polymorphisms in the moderation of the personality traits *AI* and *CI* and their role as endophenotypes of impulse-control disorders, including ADHD and SUD. More specifically, it can explain the association of D_4.7_R with impulse-control disorders. The demonstrated mediation of a stronger inhibition of cortico-striatal glutamatergic transmission mediated by D_4.7_R (in D_4.7_R knock-in mouse expressing a humanized D_4_R with the 3IL of the human D_4.7_R) (11), would then disclose a mechanism determining an increase in *AI* and *CI*.

Reviewing the results from experimental models of ADHD and RLS, it becomes evident that selective D_4_R antagonists and agonists could be respectively effective. Although a single clinical study with a D_4_R antagonist resulted negative in the treatment of ADHD (104), we believe that the reviewed preclinical evidence calls for additional clinical studies. Apart from addressing the ability of D_4_R antagonists to modify the underlying endophenotypes *AI* and *CI*, the role of D_4_R polymorphisms should also be addressed. Similarly, to our knowledge, there are no studies concerning the frequency of the different D_4_R polymorphic variants in RLS patients, and a prediction could be made about an expected lower frequency of D_4.7_R. As mentioned above, we also found a different qualitative profile of several D_2_-like receptor ligands for D_4.4_R and D_4.7_R, but only when forming heteromers with D_2_R or α_A_R (52, 53). Therefore, when searching for new D_4_R ligands, D_4_R polymorphisms and D_4_R heteromers (D_2_R-D_4_R and α_2A_R-D_4_R heteromers) should be considered as targets, which could provide a more effective and individualized treatment. Importantly, in this review we only considered the most prevalent and studied D_4_R polymorphisms, and more studies need to be performed to evaluate the specific properties of less common yet prevalent polymorphisms.

Finally, in this review we have not discussed the role of D_4_R localized in brain areas other than the frontal cortico-striatal pyramidal neuron and the pinealocytes. The globus pallidus and the lateral habenula are additional regions where D_4_Rs have been shown to play a significant role in the mediation of inhibitory transmission by dopamine or norepinephrine, respectively (120–122). The present review emphasizes the need to find the heteromeric partners of the D_4_R and to establish the differential functional and pharmacological role of its polymorphic variants.

## Author contributions

All authors listed have made a substantial, direct, and intellectual contribution to the work and approved it for publication.

## Funding

S.F. is supported by the intramural funds of the National Institute on Drug Abuse. E. M., V.C.-A. and V.C. are supported by “Ministerio de Ciencia e Innovación/Agencia Estatal de Investigación’ (MCIN/AEI/10.13039/501100011033) and FEDER (SAF2017–87629-R and PID2020–113938RB-I00). V.C.-A also acknowledges the Spanish MCIN/AEI/10.13039/501100011033 for a ‘Juan de la Cierva’ fellowship (FJC2019-041020-I).

## Conflict of interest

The authors declare that the research was conducted in the absence of any commercial or financial relationships that could be construed as a potential conflict of interest.

## Publisher’s note

All claims expressed in this article are solely those of the authors and do not necessarily represent those of their affiliated organizations, or those of the publisher, the editors and the reviewers. Any product that may be evaluated in this article, or claim that may be made by its manufacturer, is not guaranteed or endorsed by the publisher.

## Abbreviations

A, agreeableness; A_1_R and A_2A_R, adenosine A_1_ and A_2A_ receptor**;** ADHD, attention-deficit hyperactivity disorder; α_1B_R, α_2A_R and β_1_R, adrenergic α_1B_, α_2A_ and β_1_ receptor; *AI*, action impulsivity; BID, brain iron deficiency; BRET, bioluminescence resonance energy transfer; *C*, conscientiousness; *CI*, choice impulsivity; CODA-RET, complemented donor acceptor bioluminescence resonance energy transfer; *CON*, constraint; D_1_R, D_2_R, D_3_R and D_4_R, dopamine D_1_, D_2_, D_3_ and D_4_ receptor; *DRD4*, human D_4_R gene; D_4.2_R, D_4.4_R and D_4.7_R, products of *DRD4* polymorphic variants with 2, 4 and 7 tandem repeats; *E*, extraversion; GPCR, G protein-coupled receptor; 3IL, third intracellular loop; MPQ, Multidimensional Personality Questionnaire; *N*, neuroticism; *NEM*, negative emotionality; NEO-PI-R, NEO Personality Inventory-Revised; *O*, openness; *PEM*, positive emotionality; P neuron, pyramidal neuron; PV, parvalbumin; RLS, restless legs syndrome; SUD, substance use disorder.
